# Quantitative Evaluation of Stereo Visual Odometry for Autonomous Vessel Localisation in Inland Waterway Sensing Applications

**DOI:** 10.3390/s151229892

**Published:** 2015-12-17

**Authors:** Thomas Kriechbaumer, Kim Blackburn, Toby P. Breckon, Oliver Hamilton, Monica Rivas Casado

**Affiliations:** 1 School of Energy, Environmental Technology and Agrifood, Cranfield University, Cranfield MK43 0AL, UK; t.kriechbaumer@cranfield.ac.uk; 2 School of Aerospace, Transport Systems and Manufacturing, Cranfield University, Cranfield MK43 0AL, UK; k.blackburn@cranfield.ac.uk; 3 School of Engineering and Computing Sciences, Durham University, Durham DH1 3LE, UK; toby.breckon@durham.ac.uk (T.P.B.); oliver.hamilton@durham.ac.uk (O.H.)

**Keywords:** visual odometry, river monitoring, stereo vision, autonomous watercraft, survey vessel, autonomous river navigation, GPS-denied environments

## Abstract

Autonomous survey vessels can increase the efficiency and availability of wide-area river environment surveying as a tool for environment protection and conservation. A key challenge is the accurate localisation of the vessel, where bank-side vegetation or urban settlement preclude the conventional use of line-of-sight global navigation satellite systems (GNSS). In this paper, we evaluate unaided visual odometry, via an on-board stereo camera rig attached to the survey vessel, as a novel, low-cost localisation strategy. Feature-based and appearance-based visual odometry algorithms are implemented on a six degrees of freedom platform operating under guided motion, but stochastic variation in yaw, pitch and roll. Evaluation is based on a 663 m-long trajectory (>15,000 image frames) and statistical error analysis against ground truth position from a target tracking tachymeter integrating electronic distance and angular measurements. The position error of the feature-based technique (mean of ±0.067 m) is three times smaller than that of the appearance-based algorithm. From multi-variable statistical regression, we are able to attribute this error to the depth of tracked features from the camera in the scene and variations in platform yaw. Our findings inform effective strategies to enhance stereo visual localisation for the specific application of river monitoring.

## 1. Introduction

Robotics and autonomous systems are increasingly used as efficient data-gathering tools in environmental research and management and have the potential to significantly improve our capacity to monitor a wide range of environmental systems at large spatial and temporal scales [1]. In the specific context of river monitoring, autonomous survey vessels measuring physical attributes, such as water depth, velocity and discharge, can contribute towards a more efficient implementation of environmental assessments for flood protection, water supply and ecological restoration (as required by some legislation [2,3]). Conventionally, these physical river attributes are measured through hand-held mechanical meters or active acoustic sensors deployed from tethered, radio-controlled or manned platforms [4,5,6].

A key challenge to the development of autonomous river monitoring vessels is the accurate localisation of the vessel itself, required for both navigation and spatial localisation (position error <1 m) of the collected data to allow for accurate data analysis [7,8,9]. This is confounded by the natural occurrence of bank-side vegetation and urban settlement that preclude the conventional use of global positioning via line-of-sight global navigation satellite systems (GNSS; also commonly known as the Global Positioning System (GPS) when limited to the space and control segments operated by the United States Air Force) in many locales of interest. In such areas, the vessel location relative to its surrounding environment can be estimated based on range measuring on-board sensors, such as lasers, sonar or cameras [10]. Localisation based on cameras is particularly attractive, because of their relatively low cost and light weight and the ability to retrieve the environmental appearance, colour and texture, enabling the integration of high-level tasks, such as ecological feature classification [11,12].

The process of estimating the ego-motion of a robot or vehicle using the input of a single or multiple cameras attached to it is known as visual odometry [13], and the first description of vehicle navigation based solely on visual information reaches back to the work of [14] in 1980. The incremental pose estimate between camera frames is obtained based on the change in the recorded images induced by motion, and the technique relies on sufficient illumination in the environment, a static, textured scene and sufficient scene overlap between consecutive frames [15]. Based on the sensor used, monocular and stereo visual odometry can be distinguished. In this study, we only consider the latter, because techniques based on a single camera rely on additional measurements, further on-board sensors or motion constraints in order to recover the absolute scale of the camera motion (scale ambiguity problem) [13,15]. In contrast, stereo vision using calibrated cameras with a known baseline allows for the extraction of depth information with every recorded frame through triangulation. The existing visual odometry algorithms can be categorised into feature-based (sparse) and appearance-based (dense) techniques [15,16,17]. The former estimate camera poses based on the displacement of a sparse set of salient features that are detected and matched across subsequent images. These techniques involve the projection of feature points from the (2D) image domain to the (3D) real-world domain. The pose increment between frames is then commonly computed by minimising the differences between corresponding 3D feature locations from subsequent frames (absolute orientation methods) or by minimising the error in the re-projection of the transformed 3D features into the image domain (perspective in *n*-point methods). For robustness against outliers, these optimisation procedures are frequently wrapped into a random sample consensus (RANSAC) scheme [18]. Using stereo cameras, sparse visual odometry has been demonstrated on aerial and ground vehicles in a range of settings, including outdoor urban environments [19,20,21,22,23], rough terrain [17,24,25] and even extraterrestrial terrain [26]. For example, on a 9 km-long trajectory with a motorcar in rough terrain, [17] achieved a root mean square error (RMSE) in the 3D position of 45.74 m (0.49% of the trajectory), which was reduced to 4.09 m (0.04% of the trajectory) by integrating the visual odometry with angular motion estimated from an inertial measurement unit (IMU).

Dense visual odometry, on the other hand, avoids the potentially error-prone feature extraction and matching, but instead estimates the camera motion based on a direct model that involves the dense set of pixels for which depth information is available. The underpinning idea of this technique is that after the camera motion from a reference to a target frame, the re-projection of the dense cloud of previously-extracted and transformed 3D points to the image plane will yield a deformed or warped intensity image of the target frame. The solution is to find the camera pose increment that minimises a cost function based on the differences between the pixel intensities of the warped target frame and the reference frame. This optimisation has also been described as photo-consistency maximisation [27]. In [28], it was argued that minimising a cost function that is directly based on the image measurement (pixel intensities) avoids the systematic propagation of feature extraction and matching errors, reducing the resulting drift in the camera pose estimate. Dense visual odometry has found increased application with consumer depth cameras, offering co-registered colour and depth imagery (RGB-D) in indoor environments [27,28,29], but has also been applied with monocular [30] and stereo cameras in urban settings [31,32]. For a 220 m-long loop trajectory with a motorcar in a city, [32] reported an RMSE of 1.37 m (0.6% of the trajectory).

Previous studies assessing visual odometry in the inland waterway environment are rare and have focused exclusively on feature-based techniques with the dominance of aerial vehicles navigating a few metres above the water surface [33,34,35]. Stereo visual odometry in a river environment has been implemented in [34,35], who propose the fusion of a classic feature-based technique with inertial measurements from gyroscope and accelerometers and intermittent readings from a GPS device through a graph-based optimisation to correct for unbounded position drift. Although the system was designed with a focus on aerial vehicles, tests were conducted on a manned floating platform. They report a consistent under-estimation of the platform translation (by 10% on average) due to a lack of features at close range; this being a problem that is specific to certain river environments where structure is limited to the river banks. After correcting for this bias, the system (visual odometry, IMU, sparse GPS) is shown to achieve a mean position error of 5 m over a 2-km traverse [36].

In [37], it was argued that the limited reliability of existing visual odometry algorithms prevents these methods from being used for on-board guidance of a fully-autonomous vehicle in challenging environments. They emphasise the need for an increased understanding of the effect of covariates related to vehicle kinematics and scenery on the performance of existing visual odometry algorithms, in order to guide the development of more robust techniques. The challenges to robust visual odometry in the inland waterway environment arise from (i) a landscape structure that is different from that in indoor settings and urban environments, so that the specific structure of the scenery, such as orthogonality constraints and the presence of distinct corner features, cannot be assumed *a priori* [33] and (ii) from the platform kinematics, which are specific to the respective environmental monitoring application.

**Figure 1:**
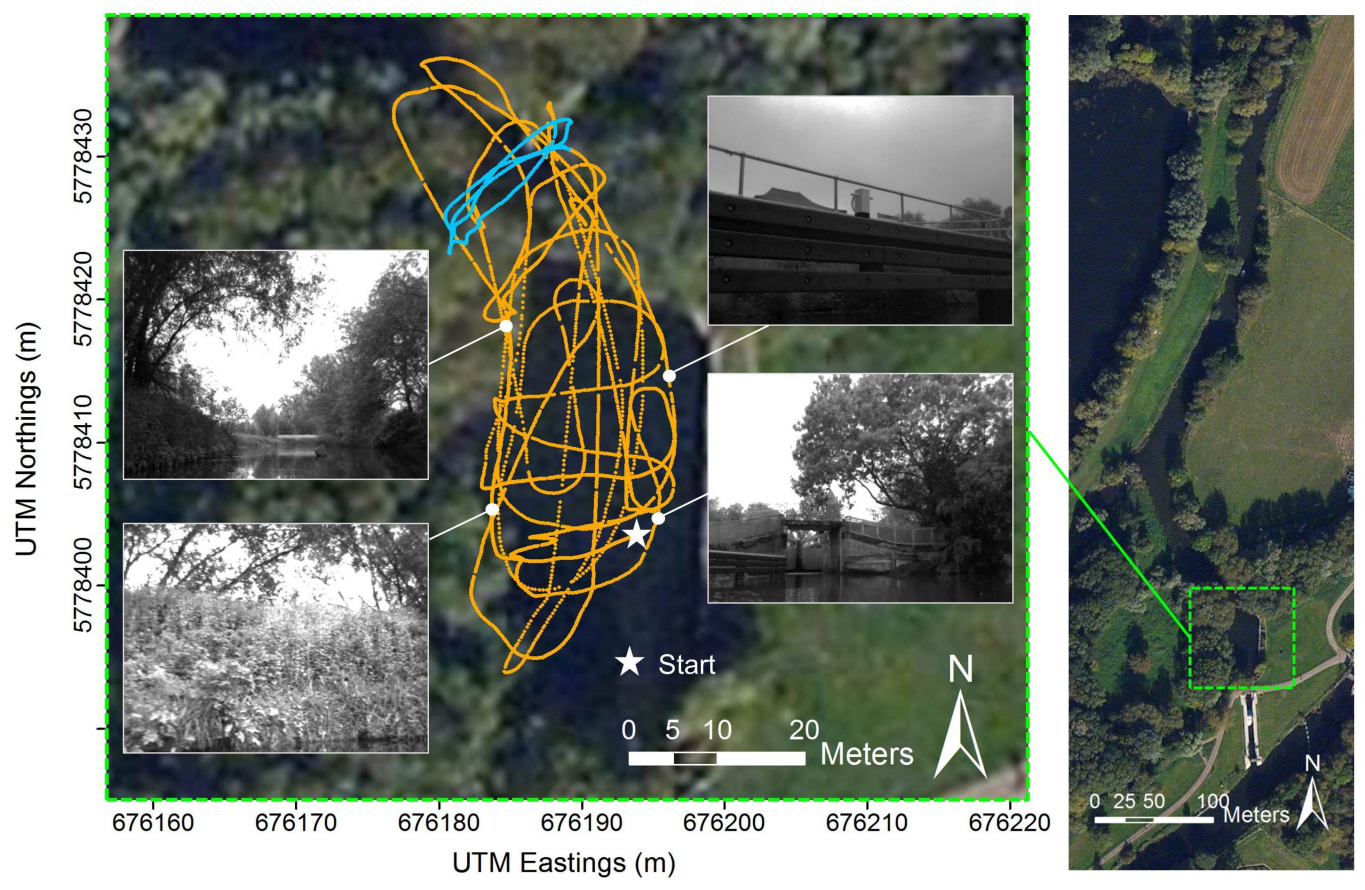
Full (orange) and discharge measurement (blue) trajectory with exemplary left intensity image samples.

Guided survey vessels equipped with active acoustic sensors to measure river discharge or to characterise the hydrodynamics of river cross-sections follow a distinct sampling strategy involving the repeated crossing of a lateral river section [4,5,38] (commonly four times or more). The vehicle operation differs from that covered in visual odometry assessment datasets in the automotive context (e.g., [39]) by the very low speed (ideally less than or equal to the average total water velocity [4]) and large changes in yaw (often with no translational motion) at the beginning and ending of crossings. The latter has been shown to be potentially detrimental to the accuracy of feature-based visual odometry due to motion blur and degeneration of the linear system to calculate the fundamental matrix [37]. Furthermore, dense visual odometry has been shown to be susceptible to errors from large camera orientation changes [32]. In addition to cross-sectional measurements, radio control survey platforms and acoustic sensors are increasingly used for surveying the river bed topography (bathymetry) and the spatial distribution of water velocities in continuous, spatially-dense sampling trajectories over small areas of interest, such as near river engineering structures [5] or over river reaches of several kilometres in length [40]. The sceneries encountered in such applications can be dominated by feature-rich, but repetitive, vegetated river banks, distant features (e.g., with the cameras pointing directly up- or down-stream on a wide river), reflections from the water surface or feature-poor engineered river structures, such as piers (see Figure 1). To be suitable for river monitoring platforms, a visual odometry system should be sufficiently robust and fast (real time) to allow for autonomous navigation given the mentioned variety of sceneries and distinct vehicle kinematics and enable spatial data referencing at accuracies similar to good quality differentially-corrected GPS in order to meet common surveying standards [7,8].

In this paper, we examine the use of unaided (*i.e.*, without integrating other sensors) visual odometry for navigating an autonomous watercraft following typical trajectories for sampling water depth, velocities and river discharge. Our study is unique because it (i) focuses on real-world river monitoring applications using established survey vessels and statistically robust data sampling strategies, (ii) assesses both feature-based and appearance-based visual odometry approaches, (iii) introduces a technique for ground truthing position estimates at accuracies of a few centimetres in truly GNSS-denied outdoor environments based on an electronic theodolite integrated with an electronic distance meter (EDM) and tracking capability (Total Station) and (iv) quantifies the error contribution of covariates related to river environment scenery and platform kinematics through multiple linear regression analysis. Thereby, we contribute to the cross-disciplinary application of techniques from the domain of mobile robotics and address the need for an increased understanding and subsequent improvement of the reliability of existing visual odometry algorithms when applied to real-world applications in challenging environments [37].

## 2. Experimental Section

### 2.1. Case Study Site

Our evaluation is based on stereo images collected along a 663 m-long trajectory (>15,000 image frames) on a 50 m-long reach of the River Great Ouse near Bedford, United Kingdom (Figure 1). This site is particularly suitable for the evaluation, because it incorporates both engineered river structures (bridges, piers) and more natural, vegetated river banks. The data collection was undertaken on 30 May 2013 at approximately 16:00 UTC, during cloudy weather conditions, a gentle breeze of wind (wind speeds of 3.4–5.4 ms^−1^) and a visibility of 9 km, according to data from the nearest U.K. Met Office weather station (Bedford). The flow conditions within the survey area were calm with total water velocities ranging from 0.00–0.24 ms^−1^ and water depths up to 3.72 m based on readings from a Teledyne RDI 1200-kHz Rio Grande acoustic Doppler current profiler (ADCP).

### 2.2. Data Collection

Grayscale stereo image frames were collected following sampling patterns realistic for bathymetric surveys of small river reaches, with a mean boat speed of 0.57 ms${}^{-1}$. The trajectory contains a sequence of four consecutive river section crossings (total length of 54 m with a mean boat speed of 0.17 ms^−1^) representative of a typical, mobile river discharge measurement (Figure 1).

**Figure 2:**
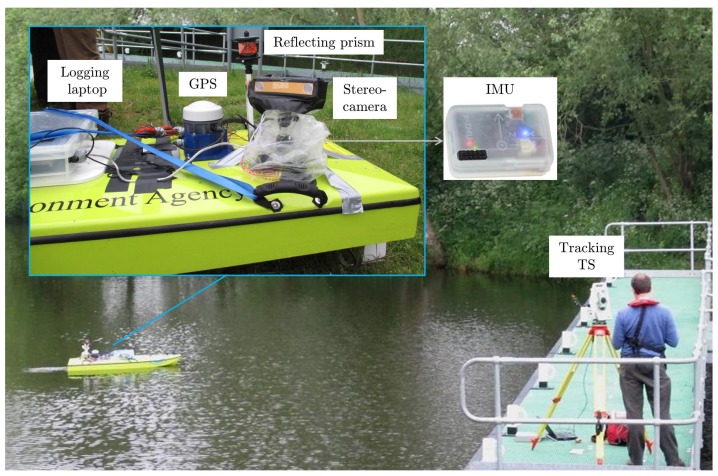
Technical data collection setup.

The platform used was a 1.95 m-long and 0.73 m-wide survey vessel (ARC-Boat; [41]), which is the most commonly-used radio controlled craft for mobile river discharge measurements within the Environment Agency of England. The platform was equipped with a Bumblebee2 stereo camera [42] with an image resolution of 1024 × 768 pixels and a stereo baseline of 0.12 m; an x-IMU inertial measurement unit fusing tri-axis MEMS gyroscopes, accelerometers and magnetometers to record pitch, roll and yaw around the sensor frame axes in Euler angles with a dynamic error <1.7 deg (root mean square) [43,44]; a GPS receiver to capture GPS performance indicators along the test trajectory, namely horizontal dilution of precision (HDOP) and the number of satellites in view, as given in the Global Positioning System Fix Data sentence (GGA) defined by the NMEA 0183 standard; and a 360° prism reflecting a modulated laser beam emitted from the electronic distance meter (EDM) of a Leica Viva TS15 Total Station [45] placed at a fixed location on the river bank (Figure 2). The TS15 is a target-tracking tachymeter integrating distance measurements (from the EDM implementing a hybrid phase shift and time of flight method [46]) and horizontal and vertical angular measurements from an electronic theodolite to provide target positions in 3D. The manufacturer-stated errors (standard deviation) are 0.003 m + 1.5 μm·m^−1^ for continuous distance measurements to a prism with a maximum tracking distance of 800 m and 1” for angular measurements [45]. In [47], the ability of tracking Total Station devices to measure target kinematics of a few millimetres was verified on a calibration track line up to distances of about 50 m. The TS15 data were transmitted to an on-board laptop via a MOXA NPort W2150 wireless device server connected to a TP-LINK 150 Mbps WiFi access point. The recording frequencies for the stereo camera, IMU, GPS and Total Station samples were 8.2 Hz (average), 128 Hz (constant), 1 Hz (constant) and 4.0 Hz (average), respectively, resulting in a total of 7491 stereo image frames with registered reference positions over the duration of the test sequence.

All data were recorded on an on-board laptop with an Intel Core2Duo 2-GHz processor using software written in C++. To enable time synchronisation of the sensors, their data were time stamped with the Windows PC time of the logging computer and temporally-aligned during data post-processing. On average, the Total Station and camera data had a temporal offset of 0.03 s, translating to a mean error in the Total Station-based reference positions of 0.02 m. The temporal offset of the IMU data was negligible due to its high recording frequency. To allow for error analysis, the Total Station and stereo camera coordinate systems were aligned using the absolute orientation algorithm of [48].

### 2.3. Visual Odometry

The platform localisation system implemented in this study is based solely on visual data from a stereo rig mounted on the back part of a survey platform and tilted sidewards from the platform centre line (line from bow to stern; Figure 2). Two fundamentally different visual odometry approaches were implemented and assessed separately: (i) a classic algorithm based on the matching of sparse features in left, right and consecutive stereo image frames that has been shown to achieve high accuracies in urban automotive applications [21] and indoor environments [37] and (ii) an appearance-based algorithm similar to that presented in [27,28] for RGB-D cameras. The inputs to the first algorithm were rectified grayscale image frames from a calibrated stereo camera. Conversely, the input to the second algorithm was stereo depth information, for co-registered grayscale pixel intensity for the scene, recovered over the same rectified image pairs via the semi-global block matching stereo approach of [49]. Rectified images were captured directly from the stereo sensor unit based on camera pre-calibration by the manufacturer.

#### 2.3.1. Using Sparse Features

**Figure 3:**
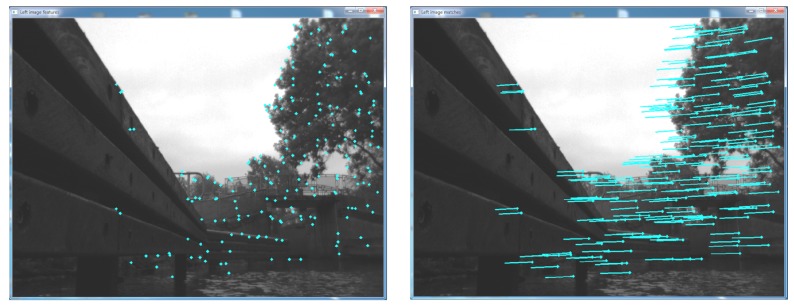
Detection (**Left**) and matching (**Right**) of sparse image features after feature reduction through bucketing.

The sparse feature-based algorithm used in this study was first introduced in [21]. Given a pair of stereo image frames with colour brightness (intensity) functions $[I^{\left(l\right)}\left(p^{\left(l\right)},t_{0}\right)$, $I^{\left(r\right)}\left(p^{\left(r\right)},t_{0}\right)]$ and $[I^{\left(l\right)}\left(p^{\left(l\right)},t_{1}\right)$, $I^{\left(r\right)}\left(p^{\left(r\right)},t_{1}\right)]$, where $p^{\left(l\right)}$ and $p^{\left(r\right)}$ are the pixels in the left and right images acquired at time *t*, the algorithm estimates the incremental camera motion from $t_{0}$ to $t_{1}$ in the following consecutive steps:

(i) detection of minimum/maximum blob and corner features in all four intensity images through image filtering with blob and corner masks of 5 × 5 pixels size (see Figure 3 in [21]) followed by non-maximum and non-minimum-suppression [50] (Figure 3);
(ii) matching of feature pairs between the intensity images by comparing the sum of absolute differences of their 11 × 11 block windows of horizontal and vertical Sobel filter responses (*i.e.*, the feature descriptor; see Figure 3 in [21] and Figure 3);
(iii) extraction of the 3D real-world position *P* of feature points in $\left[I^{\left(l\right)},I^{\left(r\right)}\right]$ as:
  (1) $$P\left(p\right)={\left(X\left(p\right)=\frac{Z\left(p\right)\;\cdot \;\left(p_{x}-c_{x}\right)}{f};\;Y\left(p\right)=\frac{Z\left(p\right)\;\cdot \;\left(p_{y}-c_{y}\right)}{f};\;Z\left(p\right)=\frac{fB}{D}\right)}^{T}$$
  where *X*, *Y* and *Z* are the real-world coordinates relative to the camera reference frame in meters, $p_{x}$ and $p_{y}$ are the coordinates of the feature points in the image domain, $c_{x}$ and $c_{y}$ are the coordinates of the image centre along the optical axis, *f* is the focal length of the camera in pixels, *B* is the stereo baseline in meters and *D* is the pixel disparity of feature pairs matched between $I^{\left(l\right)}$ and $I^{\left(r\right)}$; and
(iv) estimation of the rotation *R* and translation *T*, describing the incremental camera motion by iteratively minimising the re-projection error *e* of the extracted 3D feature points into the 2D space of $\left[I^{\left(l\right)},I^{\left(r\right)}\right]$ using Gauss–Newton optimisation, with:
  (2) $$e=\sum_{i=1}^{N}{∥p_{i}^{\left(l\right)}-\pi^{\left(l\right)}\left(P_{i};R,T\right)∥}^{2}+{∥p_{i}^{\left(r\right)}-\pi^{\left(r\right)}\left(P_{i};R,T\right)∥}^{2}$$
  whereby here, $p_{i}^{\left(l\right)}$ and $p_{i}^{\left(r\right)}$ are the feature locations in the left and right current images, respectively, and $\pi^{\left(l\right)}\left(P_{i};R,T\right)$ and $\pi^{\left(r\right)}\left(P_{i};R,T\right)$ denote the projection from 3D to 2D space.

Prior to the incremental pose estimation, the number of feature matches per 50 × 50 pixel window is reduced to two (bucketing), in order to increase the speed for real-time applications. For robustness against outliers, the minimisation procedure of the re-projection error is wrapped into a RANSAC scheme [18], where the re-projection error is independently estimated 50 times using three randomly-selected matches, and the matches of the iteration with the most inliers are used in a final optimisation to refine the estimates of *R* and *T*.

*R* and *T* are expressed as a 3 × 3 rotation matrix and a 3 × 1 translation vector, respectively, so that the incremental pose estimate becomes a 4 × 4 homogeneous transformation matrix *H* of the form $H=\left(\begin{array}{cc} R & T\\ 0 & 0 \end{array}\right)$ and the global camera pose $\hat{H}$ is updated after each frame (apart from the very first) as:

(3) $$\hat{H}⟵\hat{H}H$$

Finally, a standard Kalman filter (assuming constant acceleration) is used to smooth the estimated trajectory [51]. The state equation is formulated as:

(4) $${\left(\begin{array}{c} v\\ a \end{array}\right)}^{\left(t\right)}={\left(\begin{array}{cc} I & \Delta_{t}I\\ 0 & I \end{array}\right)}^{t}{\left(\begin{array}{c} v\\ a \end{array}\right)}^{\left(t-1\right)}+\varepsilon$$

and the output equation as:

(5) $$\frac{1}{\Delta_{t}}{\left(\begin{array}{c} R\\ T \end{array}\right)}^{\left(t\right)}=\left(\begin{array}{cc} I & 0 \end{array}\right){\left(\begin{array}{c} v\\ a \end{array}\right)}^{\left(t\right)}+\nu$$

where *v* is the velocity vector $v={\left(\begin{array}{cc} R & T \end{array}\right)}^{T}/\Delta_{t}$, $\Delta_{t}$ is the time between frames, *a* is the acceleration, *I* is the 6 × 6 identity matrix, *ε* is the process noise and *ν* is the measurement noise.

#### 2.3.2. Using Dense Features

The dense visual odometry technique implemented in this study is based on an algorithm written for RGB-D sensors [27,28] and has been adapted for the use with optical stereo image frames. As in Section 2.3.1, we start with a pair of stereo image frames with colour brightness (intensity) functions $[I^{\left(l\right)}\left(p^{\left(l\right)},t_{0}\right)$, $I^{\left(r\right)}\left(p^{\left(r\right)},t_{0}\right)]$ and $[I^{\left(l\right)}\left(p^{\left(l\right)},t_{1}\right)$, $I^{\left(r\right)}\left(p^{\left(r\right)},t_{1}\right)]$ and estimate the camera pose increment from $t_{0}$ to $t_{1}$. A disparity map can be readily obtained from inter-pixel matching of $I^{\left(l\right)}$ and $I^{\left(r\right)}$. In this study, we use semi-global block matching [49], due to its superior performance demonstrated in [52]. From this, a dense cloud of 3D points *P* is computed, as shown in Equation (1). Consider now the camera motion from the reference frame $I^{\left(l\right)}\left(p^{\left(l\right)},t_{0}\right)$ with corresponding 3D points $P(p,t_{0})$ to the target frame $I^{\left(l\right)}\left(p^{\left(l\right)},t_{1}\right)$, expressed as a 4 × 4 homogeneous transformation matrix *H* of the form $H=\left(\begin{array}{cc} R & T\\ 0 & 0 \end{array}\right)$ , where *R* is a 3 × 3 rotation matrix and *T* is a 3 × 1 translation vector. The transformation G(H,P) of the 3D points $P(p,t_{0})$ corresponding to the camera motion is:

(6) G(H,P)=RP+T

The transformed 3D points are re-projected to the image domain of the target frame using the projection *π*:

(7) $$\pi \left(G\right)={\left(\frac{G_{1}+f}{G_{3}}-c_{x}\;,\;\frac{G_{2}+f}{G_{3}}-c_{y}\right)}^{T}$$

This yields a warped intensity image of the target frame, which can generally be described through the so-called warping function [28] or image warp [27] as:

(8) $$\varpi \left(p,t\right)=\pi \left(G(H,P(p))\right)$$

Using the Levenberg–Marquardt method, the algorithm finds the camera pose transformation *H* that minimises the non-linear least-squares cost function E(H):

(9) $$E\left(H\right)=\sum_{p}{\left[I\left(\varpi \left(p,t_{1}\right),t_{1}\right)-I\left(p,t_{0}\right)\right]}^{2}$$

**Figure 4:**
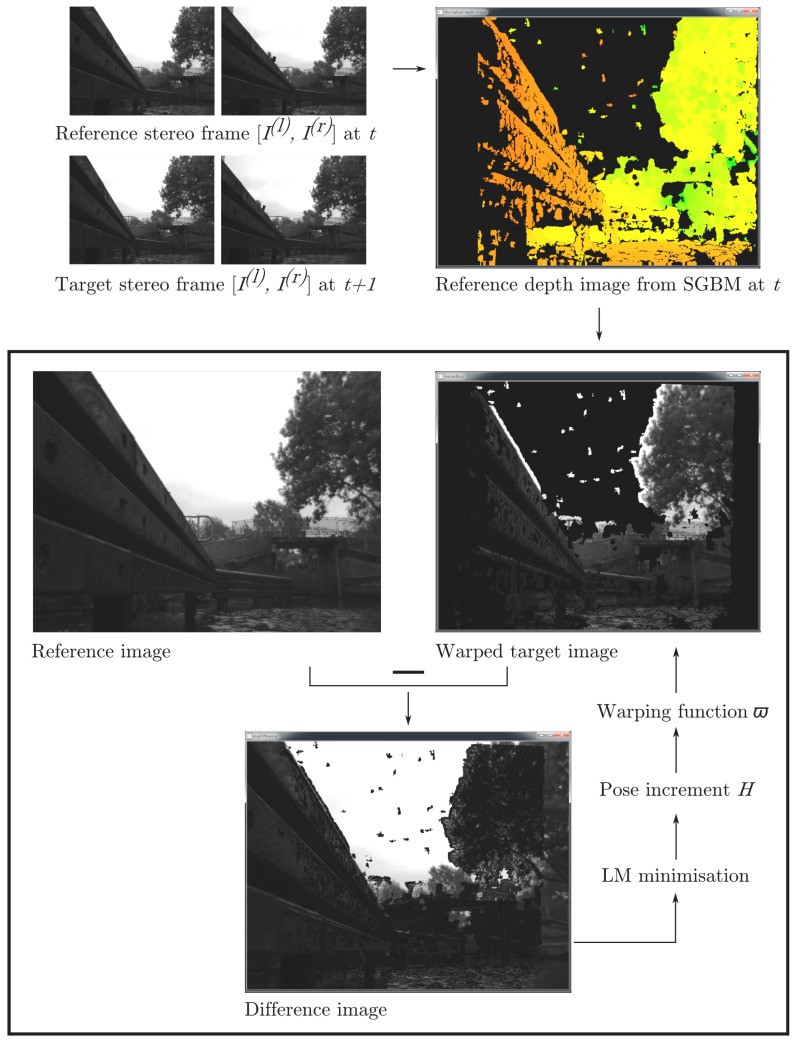
Illustration of the dense visual odometry algorithm for the final image pyramid level [27,28]; SGBM and LM stand for semi-global block matching and Levenberg–Marquardt, respectively.

A conceptual flow diagram of the dense visual odometry algorithm is given in Figure 4.

We implement the optimisation using a multi-resolution approach as suggested in [32], where the camera pose transformation is iteratively estimated on the levels of an image pyramid encompassing four image resolutions. The algorithm starts with the lowest resolution to obtain a first estimate of *H*, which is then used to initialise the subsequent minimisation applied to the next highest resolution, and so on, until the resolution of the original images is reached. This approach increases the computational efficiency, because smaller images are used to perform the minimisation for larger frame to frame motion, whereas the minimisation with the larger images serves to refine the transformation estimate [32]. The maximum number of iterations was set to 40, 20, 3 and 1, for the four scales from coarsest to finest. The decimated images are generated using a simple bi-linear interpolation method with scaling factors of 0.125, 0.25, 0.5 and 1, for the four pyramid levels. To eliminate aliasing induced by image re-sampling, the downscaled intensity images are blurred using a Gaussian filter with a kernel standard deviation of three in the *x* and *y* directions.

From the estimated pose increments, the global pose trajectory is obtained as in Equation (3).

### 2.4. Validation

Both visual odometry algorithms were quantitatively evaluated regarding their pose estimation accuracy and their robustness in a variety of sceneries and vehicle kinematics. Our evaluation approach builds on that in [17], who presented a valid framework to separately quantify the random and systematic error components of the 6D pose estimation from visual odometry. The analysis follows an odometry model consisting of translation and angular errors over displacement. The translation errors $\varepsilon_{m}$ and the angular errors $\varepsilon_{n}$ are computed as:

(10) $$\varepsilon_{m}=m_{TS}-m_{VO}\;\;\;\forall m=\left\{x,y,z\right\}$$

and:

(11) $$\varepsilon_{n}=n_{IMU}-n_{VO}\;\;\;\forall n=\left\{\phi ,\theta ,\psi \right\}$$

where $m_{TS}$, $m_{VO}$ are the camera translation parameters measured by the Total Station and estimated through visual odometry and $n_{IMU}$, $n_{VO}$ are the rotation parameters measured by the IMU and estimated through visual odometry; {x,y,z} are the sensor frame axes (*y* points along the camera optical axis, and we follow a right-hand coordinate system); and {ϕ,θ,ψ} are the pitch, roll and yaw rotations about these axes in Euler angles. The total position error in 3D space $\varepsilon_{p}$ is computed as:

(12) $$\varepsilon_{p}=\sqrt{{\varepsilon_{x}}^{2}+{\varepsilon_{y}}^{2}+{\varepsilon_{z}}^{2}}$$

Following [17], we divide the test trajectory into 100 consecutive sections of an approximately 6.5-m length each. The length of 6.5 m is meaningful given the very low platform speeds and the context of monitoring small rivers. For each section, the position drifts in the *x*, *y* and *z* direction, and the angular drifts about each of these axes (in Euler angles) are integrated. The errors over displacement (denoted $\varepsilon_{m}^{{}^{\prime }}$, $\varepsilon_{n}^{{}^{\prime }}$ and $\varepsilon_{p}^{{}^{\prime }}$) are computed by dividing the respective error ($\varepsilon_{m}$, $\varepsilon_{n}$, $\varepsilon_{p}$) at the end of the section by the section length.

We formulate a multiple linear regression model to explain the position error over displacement $\varepsilon_{p}^{{}^{\prime }}$ for the sample of 6.5 m-long sections indexed by *i*, using covariates related to scenery and platform kinematics:

(13) $$\varepsilon_{p,i}^{{}^{\prime }}=\beta_{0}+\beta_{1}v_{i}+\beta_{2}h_{i}+\beta_{3}f_{i}+\beta_{4}d_{i}+u_{i}$$

where the covariates are:

- *v*: the section mean of total vessel speed (in ms^−1^), quantified as distance travelled over time based on the Total Station measurements;
- *h*: the section variability in platform yaw (in deg), quantified as the standard deviation of the platform yaw measured by the IMU;
- *f*: the section mean number of inlier feature matches (for sparse visual odometry) or the number of pixels with valid depth information (for dense visual odometry); and
- *d*: the section mean depth (in m) of matched inlier features (for sparse visual odometry) or the mean depth of pixels with valid depth information (for dense visual odometry).

The partial regression coefficients $\beta_{1}$, $\beta_{2}$, $\beta_{3}$ and $\beta_{4}$ describe the marginal contribution of the associated covariates to variations in $\varepsilon_{p}^{{}^{\prime }}$ provided that all other covariates are held constant. *u* is the model error capturing differences in the predicted and observed values (also referred to as residuals) of $\varepsilon_{p}^{{}^{\prime }}$. The partial regression coefficients were estimated using ordinary least squares (OLS) and the assumptions for linear regression with OLS (*i.e.*, normally-distributed residuals $u∼N(0,\sigma^{2})$, uncorrelated and homoskedastic residuals and lack of multicollinearity between the covariates) were assessed via residual and correlation analysis. The statistical significance of the coefficients was tested through *t*-tests (α=0.05), and the overall model validity was assessed through F-tests (α=0.05). For a detailed outline of multiple linear regression analysis, the reader is referred to [53].

Moreover, to evaluate the visual odometry algorithms over longer trajectories, we compute the maximum and RMSE of $\varepsilon_{p}$ from all registered position estimates *N* of the 663 m-long trajectory, as well as for the sub-trajectory covering the discharge measurement (repeated river section crossing) only. The RMSE is computed as:

(14) $$RMSE=\sqrt{\frac{1}{N}\sum_{j=1}^{N}\varepsilon_{p,j}^{2}}$$

## 3. Results and Discussion

### 3.1. Error Statistics

**Figure 5:**
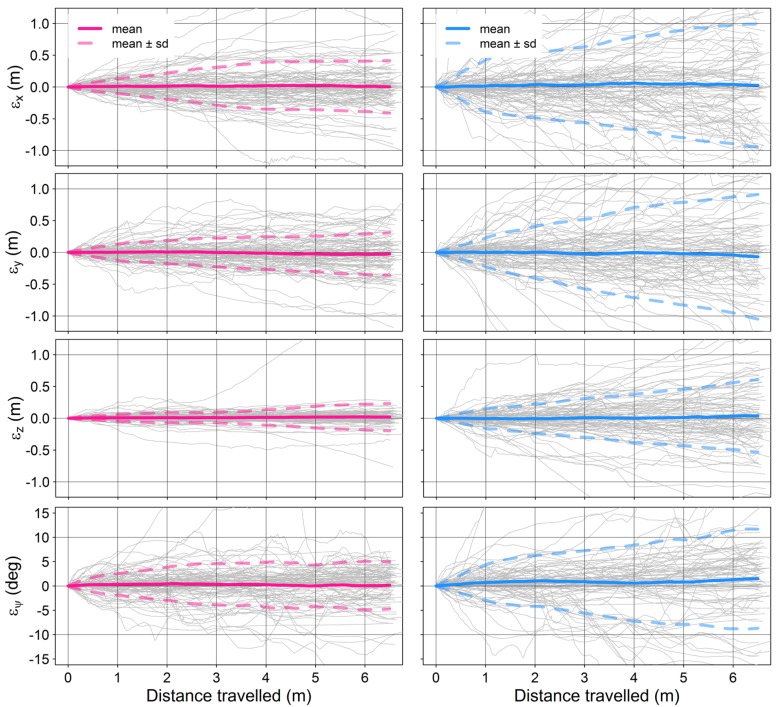
Translation and angular errors (shown for yaw only) over a sample of approximately one hundred 6.5 m-long trajectories for sparse (left column) and dense (right column) visual odometry.

Table 1 shows the statistical distributions of the 3D position, translation and angular errors over displacement for both visual odometry techniques assessed. With a magnitude of 0.067 m·m^−1^, the mean position error per metre displacement of the sparse visual odometry technique is almost three times lower than that of the dense technique. The translation errors of both techniques show a mean very close to zero, suggesting that the error is not systematic over the trajectory distance evaluated. The errors in the estimated pitch and roll are considerable with magnitudes up to nearly 4 deg·m^−1^ (for sparse) and larger than 8 deg·m^−1^ (for dense). Figure 5 illustrates the error accumulation over the course of the 6.5 m-long sub-trajectories.

**Table 1:** 3D position and translation error over displacement (in m·m^−1^) and angular errors over displacement (deg·m^−1^) for sparse and dense visual odometry, computed from *n* sections of an approximately 6.5-m length each.

		Min	Mean	Median	SD	Max	*n*
sparse	$\varepsilon_{p}^{{}^{\prime }}$	0.004	0.067	0.048	0.060	0.345	
	$\varepsilon_{x}^{{}^{\prime }}$	−0.197	0.001	−0.003	0.064	0.229	
	$\varepsilon_{y}^{{}^{\prime }}$	−0.180	−0.002	−0.005	0.054	0.209	
	$\varepsilon_{z}^{{}^{\prime }}$	−0.117	0.002	0.002	0.033	0.263	
	$\varepsilon_{\phi }^{{}^{\prime }}$	−4.16	−0.01	0.07	1.39	3.87	
	$\varepsilon_{\theta }^{{}^{\prime }}$	−3.95	0.00	0.03	1.25	3.13	
	$\varepsilon_{\psi }^{{}^{\prime }}$	−3.44	0.03	0.13	0.70	2.24	96
dense	$\varepsilon_{p}^{{}^{\prime }}$	0.007	0.177	0.139	0.149	0.757	
	$\varepsilon_{x}^{{}^{\prime }}$	−0.563	0.002	−0.002	0.151	0.564	
	$\varepsilon_{y}^{{}^{\prime }}$	−0.755	−0.010	−0.002	0.152	0.505	
	$\varepsilon_{z}^{{}^{\prime }}$	−0.366	0.005	−0.000	0.089	0.278	
	$\varepsilon_{\phi }^{{}^{\prime }}$	−7.10	−0.14	−0.14	2.84	6.54	
	$\varepsilon_{\theta }^{{}^{\prime }}$	−8.48	0.10	0.05	2.94	8.46	
	$\varepsilon_{\psi }^{{}^{\prime }}$	−5.27	0.23	0.40	1.51	4.20	92

**Figure 6:**
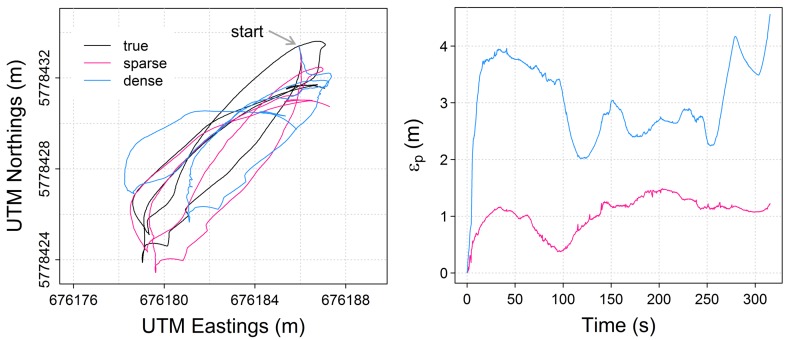
Position estimates (**Left**) and accumulation of 3D position error $\varepsilon_{p}$ (**Right**) for a discharge measurement track of four consecutive river crossings.

**Figure 7:**
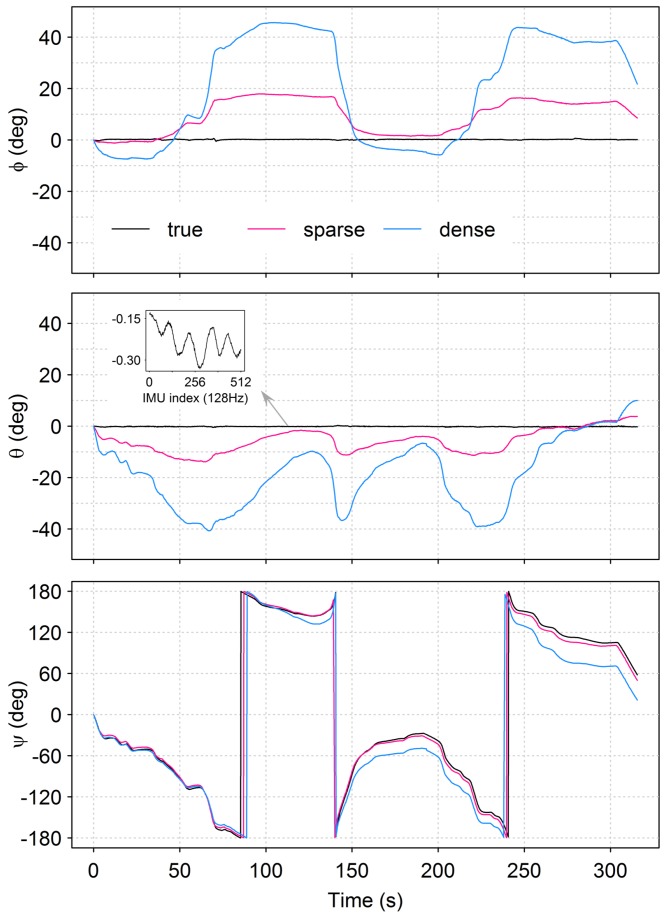
Camera orientation estimates in Euler angles for a discharge measurement track of four consecutive river crossings; *ϕ*, *θ* and *ψ* stand for pitch, roll and yaw, respectively.

Figure 6 and Figure 7 illustrate the performance of visual odometry over the 54 m-long discharge measurement trajectory involving four consecutive river crossings. As is typical in vessel-based discharge measurements, the trajectory starts with a large rotation about the vertical axis to bring the platform into position for the first river crossing. This rapid change in yaw might explain the large error accumulation in both visual odometry techniques at the beginning of the trajectory. At the end of the four crossings, the 3D position estimates of the sparse and dense techniques have drifted by 1.20 m and 4.56 m, respectively, from the Total Station-based ground truth platform position. Given the flat water surface across the measurement section, it is reasonable to take into account only the positions in 2D, in which case the sparse technique achieves an error of 0.81 m at the end of the trajectory and an RMSE of 0.83 m for the full discharge measurement track (Table 2). As illustrated in Figure 7, both visual odometry techniques keep track of the frequently-changing platform yaw, but estimate pitch and roll magnitudes and patterns without apparent relation to the true platform pitch and roll with amplitudes, typically ≪1deg (see the inlay plot in Figure 7). This leads to a maximum error in the pitch and roll estimates of 17.78 deg and 13.88 deg for the sparse, as well as 45.48 deg and 40.56 deg for the dense technique, over the course of the discharge measurement trajectory (Figure 7).

**Table 2:** Root mean square error (RMSE) and maximum of the 3D and 2D position errors, for the discharge measurement track of four consecutive river crossings and the full test trajectory; errors are given in m and % of track length (in brackets); *N* stands for the sample size of registered stereo image frames.

		3D		2D		
		RMSE	max	RMSE	max	N
Discharge measurement	sparse	1.08 (1.99)	1.51 (2.80)	0.83 (1.54)	1.21 (2.25)	
	dense	3.10 (5.74)	4.56 (8.44)	1.94 (3.58)	3.08 (5.69)	1265
Total trajectory	sparse	13.36 (2.01)	25.01 (3.77)	9.56 (1.44)	18.03 (2.72)	
	dense	31.49 (4.75)	65.43 (9.87)	21.53 (3.25)	56.83 (8.57)	7491

The error statistics for the discharge measurement and the full test trajectory are shown in Table 2. While the sparse visual odometry technique overestimates the total trajectory length slightly by 14.06 m (2.1% of the true trajectory length), the dense technique results in an under-estimation of the trajectory length by 40.70 m (6.1%).

### 3.2. Effects of Platform Kinematics and Scenery

The multiple linear regression models explain 56% and 25% of the variability in the 3D position error over displacement for the sparse and the dense visual odometry technique, respectively (see the respective measure of determination $R^{2}$ values in Table 3). In the case of sparse visual odometry, statistically-significant effects on $\varepsilon_{p}^{{}^{\prime }}$ are found only for the section variability in platform yaw *h* and the section mean depth of matched inlier features *d* (Table 3). In contrast, the linear model of the dense visual odometry error shows a statistically-significant effect of the mean vessel speed *v*, but no effect of the platform yaw variability. Here, the effect of *d* is also significant. The distributions of the covariates are presented in Table 4 illustrating the spectrum of platform kinematics and feature/depth point abundance and depth covered. The residual analysis revealed evidence for heteroscedastic residuals (all other model assumptions are fulfilled). While this does not affect the unbiasedness of the coefficient estimators, it means that the *t*- and F-statistics cannot be assumed to be exact. This could limit the predictive use of the model, but does not compromise the explanatory purpose of our analysis, *i.e.*, to explain the visual odometry errors in order to inform further improvements of the technique.

**Table 3:** Multiple linear regression model coefficients and predictive power for both sparse and dense visual odometry; * marks the statistical significance at α=0.05.

		*β*	*t*	*p*-Value (*t*)	F	*p*-Value (F)	*R*${}^{2}$
sparse	Intercept	-2.23e-02	0.82	0.42			
	*v*	5.22e-04	0.04	0.97			
	*h*	2.33e-03	9.53	0.00 *			
	*f*	6.84e-05	1.12	0.27			
	*d*	1.46e-03	2.73	0.01 *	28.70	0.00 *	0.56
dense	Intercept	-3.51e-02	−0.20	0.85			
	*v*	1.24e-01	2.06	0.04 *			
	*h*	1.39e-03	1.24	0.22			
	*f*	-1.08e-07	−0.43	0.67			
	*d*	9.84e-03	3.52	0.00 *	7.10	0.00 *	0.25

**Table 4:** Statistical distribution of multiple linear regression model covariates for both sparse and dense visual odometry.

		Min	Mean	Median	SD	Max	*n*
sparse	*v* (ms${}^{-1}$)	0.10	0.57	0.53	0.37	1.49	
	*h* (deg)	0.33	17.42	10.45	18.37	75.08	
	*f*	40	233	238	90	422	
	*d* (m)	4.13	21.60	20.53	9.30	39.38	96
dense	*v* (ms${}^{-1}$)	0.10	0.56	0.53	0.35	1.42	
	*h* (deg)	0.33	17.79	11.13	18.48	75.08	
	*f*	306,400	540,700	568,000	88,598	656,400	
	*d* (m)	6.13	20.75	20.03	7.90	36.85	92

The proportion of variability in the positioning error that remains unexplained by the regression models can be due to model misspecifications (e.g., omitted explanatory variables) or inherent stochasticity in the data generation process of $\varepsilon_{p}^{{}^{\prime }}$ [53]. The latter is grounded in the uncertainty of the positions of image points or features, which propagates to the stages of stereo matching and point/feature extraction to 3D, incremental pose estimation and, finally, the absolute camera pose estimate [22]. Further potential error sources are the absolute orientation algorithm to align the Total Station and camera coordinate systems [20] and the time synchronisation of these two sensors [5].

### 3.3. Implications for Autonomous River Monitoring

Our results indicate that unaided visual odometry can become a useful technique for localising river monitoring vessels in GPS-denied areas. Over short trajectories, such as cross-sectional sampling for discharge measurements in small rivers, a classic feature-based algorithm using off-the-shelf stereo cameras was shown to achieve 2D position errors (RMSE) below 1 m (Table 2). The magnitudes of random errors encountered for estimating platform yaw (Table 2) are comparable to those of calibrated fluxgate compasses that are integrated in many acoustic sensors to relate the measured 3D water velocity vector to the magnetic north [4]. Feature-based visual odometry provides sufficiently accurate yaw data to substitute fluxgate compass readings in areas where these are biased by magnetic interference (e.g., from steel sheet pilings or steel-reinforced river banks) and, as such, offers a low-cost alternative to GPS compasses or IMUs [5,54]. To enable vessel localisation over larger trajectories during wide-area river monitoring or bathymetric surveying of river reaches, the visual odometry techniques assessed require further improvements to limit the position error.

The large errors found in the vessel pitch and roll estimates indicate that techniques, such as the integration of on-board inertial sensors, will be essential to constrain the estimated platform pitch and roll. In [17], it was shown that even low-cost IMUs can improve the position accuracy of visual odometry by more than an order of magnitude. The position error could be reduced further by implementing the so-called loop-closure technique, which minimises drift by identifying when a previously seen scenery is revisited [55,56]. This technique might prove particularly useful in reach-wide bathymetric or hydrodynamic surveys involving numerous closed trajectory loops.

For sparse visual odometry, the error was shown to increase with the variability of vessel yaw. The 50% of the sub-trajectory samples with the lowest yaw variability show a mean 3D position error over displacement of 0.032 m·m^−1^, whereas the 50% with the largest variability have a mean error of 0.100 m·m^−1^. This sensitivity was not found for dense visual odometry, where the mean error of samples with large yaw variability (0.194 m·m^−1^) is similar to that for samples with low variability (0.215 m·m^−1^). Those sub-trajectory samples for which the dense algorithm has a lower 3D position error over displacement than the sparse technique (22 in total) were mainly such with a relatively large yaw variability, with 10 of them belonging to the quarter of samples with the largest yaw variability (>25.97 deg). While the dense technique requires further improvement to reduce its position error overall, this contrast in the sensitivity to yaw variability could be exploited in a hybrid visual odometry technique that switches between a sparse and a dense algorithm based on platform kinematics. A general improvement of the dense algorithm might be achieved by implementing a quadrifocal geometry model [32], rather than assuming that pixel locations in the depth image correspond to pixel locations in the left intensity image of the stereo pair ($p_{L}\approx p$). Moreover, in contrast to the sparse technique, the dense algorithm does not include a trajectory smoothing Kalman filter, which limits the direct comparison of the two techniques in this study.

The increase of the position error with the distance of objects in view relative to the camera can be explained by the uncertainty in these object’s 3D positions derived from stereo matching. This uncertainty increases quadratically with the distance and is inversely proportional to the stereo baseline [22,57]. A larger baseline can reduce this effect, e.g., for a distance of 20 m, a baseline increase from 12–50 cm reduces the standard deviation of the object’s displacement error from 0.23 m–0.06 m, assuming the disparity estimation error empirically found in [57]. The under-estimation of the trajectory length as reported in [35] was not confirmed in this study, which might be explained by the small scale of our case study site, assuring a sufficient number of features at close range during most times. No statistically-significant effect on the error over displacement was found for the number of RANSAC inlier features. The lowest feature number (40; see Table 4) occurred when moving along a relatively unstructured pier on site (see the corresponding sample intensity image in Figure 1), but even there, the position error was not found to be increased relative to the mean position error. Moreover, we found no evidence of large errors caused by violations of the static scene assumption (e.g., moving animals or vegetation), which shows that (for the specific conditions during the test data collection), the RANSAC-based detection of outlier feature matches and the simple Kalman filter were sufficient to overcome these potential error sources. Further studies are required to assess the effect of windier conditions causing a more dynamic scenery and larger turbulence causing higher vessel pitch and roll amplitudes on the visual odometry performance.

The data on GPS performance indicators collected with the camera frames confirm the need for vessel localisation solutions independent from navigation satellites. During 46.4% of the data collection time, the number of GPS satellites in view was lower than the minimum of four satellites required to obtain a position solution. The median number of satellites in view was four and for those samples with a valid solution the mean HDOP was 2.8 (values ≤1 indicate an ideal satellite geometry). The degradation or complete lack of GNSS-based localisation is a common and yet unsolved problem in vessel-based river monitoring. The issue has been reported for sites ranging from creeks in heavily-wooded deep valleys [58], to large rivers, where high vegetation or buildings complicate data collection in ecologically-relevant areas near the river banks [59]. Our study illustrates that visual odometry can become an important method to overcome this issue and to extend the applicability of vessel-based river monitoring to a larger number of sites.

Floating survey vessels equipped with cameras create opportunities in river monitoring that go beyond localisation. In previous research, optical imagery from aerial vehicles has been shown to enable the efficient, automated characterisation of physical in-stream and riparian ecosystem features [11,12,60]. The imagery collected from cameras on survey vessels might complement such analyses and allow for detailed, ground-based monitoring of erosion, vegetation and hydraulic patterns. The integration of active acoustic sensors measuring water depth and velocity and cameras on a single monitoring vessel has large potential for a holistic, *i.e.*, below and above the water surface, physical assessment of river environments.

## 4. Conclusions

Feature-based and appearance-based visual odometry algorithms have been evaluated for localising a river survey vessel monitoring discharge, bathymetry and hydrodynamics in a widely GPS-denied river reach. The feature-based technique was shown to perform sufficiently well for localisation over short trajectory surveys of cross-sectional hydrodynamics or discharge measurements. Multiple linear regression analysis revealed that the error is driven by variation in platform yaw and the depth of features in the scenery relative to the camera. Large errors were encountered in the platform pitch and roll estimates. These findings enable the design of effective strategies to enhance stereo visual odometry algorithms for the specific application of river monitoring. Besides platform localisation, further studies are required to explore the usefulness of on-board stereo cameras for vision-based river bank monitoring from floating vessels.
